# HMGB1 Promotes Mitochondrial Dysfunction–Triggered Striatal Neurodegeneration via Autophagy and Apoptosis Activation

**DOI:** 10.1371/journal.pone.0142901

**Published:** 2015-11-13

**Authors:** Lin Qi, Xue Sun, Feng-E Li, Bao-Song Zhu, Frank K. Braun, Zhi-Qiang Liu, Jin-Le Tang, Chao Wu, Fei Xu, Hui-Han Wang, Luis A. Velasquez, Kui Zhao, Feng-Rui Lei, Ji-Gang Zhang, Yun-Tian Shen, Jian-Xuan Zou, Hui-Min Meng, Gang-Li An, Lin Yang, Xing-Ding Zhang

**Affiliations:** 1 Hematology Center, Cyrus Tang Medical Institute, Soochow University, Suzhou, Jiangsu, China; 2 Department of Lymphoma/Myeloma, The University of Texas MD Anderson Cancer Center, Houston, TX, United States of America; 3 Laboratory of Molecular Neuro-Oncology, Texas Children's Hospital, Baylor College of Medicine, Houston, TX, United States of America; 4 Department of Emergency, First Affiliated Hospital of Soochow University, Suzhou, Jiangsu, China; 5 Department of Interventional Treatment, Tianjin Medical University Cancer Hospital and Institution, Laboratory of Cancer Prevention and Therapy, Tianjin, China; 6 Department of General Surgery, The Second Affiliated Hospital of Soochow University, Suzhou, Jiangsu, China; 7 Department of Vascular Surgery, The Second Affiliated Hospital of Soochow University, Suzhou, Jiangsu, China; 8 Department of Radiotherapy Oncology, The Second Affiliated Hospital of Soochow University, Suzhou, Jiangsu, China; The University of Texas MD Anderson Cancer Center, UNITED STATES

## Abstract

Impairments in mitochondrial energy metabolism are thought to be involved in many neurodegenerative diseases. The mitochondrial inhibitor 3-nitropropionic acid (3-NP) induces striatal pathology mimicking neurodegeneration *in vivo*. Previous studies showed that 3-NP also triggered autophagy activation and apoptosis. In this study, we focused on the high-mobility group box 1 (HMGB1) protein, which is important in oxidative stress signaling as well as in autophagy and apoptosis, to explore whether the mechanisms of autophagy and apoptosis in neurodegenerative diseases are associated with metabolic impairment. To elucidate the role of HMGB1 in striatal degeneration, we investigated the impact of HMGB1 on autophagy activation and cell death induced by 3-NP. We intoxicated rat striata with 3-NP by stereotaxic injection and analyzed changes in expression HMGB1, proapoptotic proteins caspase-3 and phospho-c-Jun amino-terminal kinases (p-JNK). 3-NP–induced elevations in p-JNK, cleaved caspase-3, and autophagic marker LC3-II as well as reduction in SQSTM1 (p62), were significantly reduced by the HMGB1 inhibitor glycyrrhizin. Glycyrrhizin also significantly inhibited 3-NP–induced striatal damage. Neuronal death was replicated by exposing primary striatal neurons in culture to 3-NP. It was clear that HMGB1 was important for basal autophagy which was shown by rescue of cells through HMGB1 targeting shRNA approach.3-NP also induced the expression of HMGB1, p-JNK, and LC3-II in striatal neurons, and p-JNK expression was significantly reduced by shRNA knockdown of HMGB1, an effect that was reversed by exogenously increased expression of HMGB1. These results suggest that HMGB1 plays important roles in signaling for both autophagy and apoptosis in neurodegeneration induced by mitochondrial dysfunction.

## Introduction

Exposure to 3-nitropropionic acid (3-NP), an irreversible inactivator of succinate dehydrogenase, induces striatal neural damage in the caudate/putamen. This was shown in humans as well as in animal experiments [1,2]. Impairments in mitochondrial energy metabolism are thought to be involved in most neurodegenerative diseases, and 3-NP–mediated impairment of cellular energy levels resembles/mimics key pathophysiological features of neurodenerative diseases, including preferential striatal degeneration such as that in Huntington disease [1,2]. Recent studies showed, moreover, increased death of neuronal cells exposed to 3-NP, with both apoptotic and necrotic features [3]. However, the exact molecular mechanisms underlying such mixed forms of cell death are not fully understood.

Increasing evidence points to a complex interplay between autophagy and apoptotic cell death signaling mediated by 3-NP [4,5]. Our and other groups showed that 3-NP triggers p53-dependent autophagy as well as cell death [6]. In addition to activation of p53, a key tumor suppressor protein, 3-NP alters expression of several apoptosis-regulating proteins of the Bcl-2 protein family [7]. Among the relevant 3-NP modulated mediators are Bcl-2–associated X protein (Bax), p53-upregulated modulator of apoptosis (PUMA), and damage-regulated autophagy modulator (DRAM), which is known to regulate autophagic flux through lysosome permeability [7–9].

High-mobility group box 1 (HMGB1) protein is a chromatin-binding nuclear protein that is part of a damage-associated molecular pattern and is important for oxidative stress response as well as for cell death signaling, including autophagy and apoptosis. Interaction of p53 with HMGB1 in response to DNA damage promotes binding of HMGB1 to damaged DNA and thereby provides a molecular platform for subsequent p53-mediated DNA repair [10]. Interestingly HMGB1 was also shown to respond to other cell stresses and display antiapoptotic effects, which were thought to be due to the capacity of HMGB1/p53 to cross-regulate/switch between apoptosis and autophagy in different cell stress settings [11]. In leukemia cells, HMGB1 was shown to be a key player in direct activation of autophagy involving activation of the PI3KC3-MEK-ERK pathway [12]. Increased expression of HMGB1 protein enhanced the transcriptional activity of c-Jun amino-terminal kinase (JNK) and extracellular signal-regulated kinase (ERK) signaling, and vice versa inhibition of HMGB1 blocked the transcriptional activities of JNK and ERK significantly[13].

In this study, we investigated, in primary striatal neurons and in rats, the impact of HMGB1 on autophagy and cell death signaling under metabolic stress conditions (3-NP). Our results suggest that HMGB1 expression, in conjunction with JNK signaling, is central in 3-NP–induced signaling. Furthermore, we show that elevated levels of HMGB1 have a neuroprotective capacity that might be relevant for novel therapeutic approaches in neurodegenerative diseases.

## Materials and Methods

### Stereotaxic drug administration

Drugs were administered stereotaxically as described previously [6]. Sprague-Dawley rats (280–300g) were obtained from the Center for Experimental Animals, Soochow University (Certificate No. 20020008, Grade II). Prior to surgery, rats were anesthetized with 4% chloralhydrate (400 mg/kg body weight). Drugs were infused into the left striatum via a cannula under stereotaxic guidance (Kopf stereotaxis, Harvard Apparatus, Holliston, MA) as described by Qin *et al*. [14]. The coordinates were: AP = 1.0 mm anterior to Bregma, ML = 2.5 mm from midline, DV = 5.4 mm below the dura. 3-NP was dissolved in isotonic saline solution with pH adjusted to 7 [5]. Glycyrrhizin was dissolved in isotonic phosphate-buffered saline solution (PBS). Striatal tissue was dissected and total RNA and proteins was extracted for real-time PCR and western blot analysis, respectively. The animal surgery protocols were approved by the Institutional Animal Care and Use Committee (IACUC) of Soochow University. Animals were maintained and all animal experiments were carried out according to the Animal Care Guidelines of Soochow University. Mice were euthanized by cervical dislocation.

### Quantitative real-time PCR

Quantitative real-time PCR was performed as described previously [7]. Total RNA was extracted from the dissected striata with the RNAiso Reagent kit (Takara, DaLian, China). cDNA was generated by reverse-transcription of 2 μg total RNA using random primers and Primescript RT Reagent Kit (Takara) in a total reaction volume of 20 μL according to the manufacturer’s instructions. The sequences of forward and reverse oligonucleotide primers used for amplification of specific gene sequences of *HMGB-1* (forward primer: 5′-ATG GGC AAA GGA GAT CCT A-3′; reverse primer: 5′-ATT CAT CAT CAT CAT CTT CT-3′) and glyceraldehyde 3-phosphate dehydrogenase (*GAPDH*; forward primer: 5′-TCC CTC AAG ATT GTC AGC AA-3′; reverse primer: 5′-AGA TCC ACA ACG GAT ACA TT-3′). Quantitative real-time quantitative PCR was performed with iCycler 5 (Bio-Rad, Hercules, CA). A 20-fold dilution of each cDNA was amplified in a 20-μL volume using the RT² qPCR Primer Assay (QIAGEN Inc., Valencia, CA), with 200 nM final concentrations of each primer. PCR cycle conditions were 95°C for 10 sec and 50 cycles of 9°C for 20 sec and 60°C for 30 sec. The amplification specificity was evaluated with melting curve analysis. Threshold cycle values, Ct, which correlates inversely with the target mRNA levels, were calculated with the second derivative maximum algorithm provided by the iCycler software. For each cDNA, the mRNA levels were normalized to *GAPDH* mRNA levels.

### Western blot analysis

Western blot analysis was performed as described previously [15]. Primary antibodies used were HMGB1 (3935, polyclonal, Cell Signaling Technology, Danvers, MA), phospho-SAPK/JNK (Thr183/Tyr185, 9251, Cell Signaling Technology), SAPK/JNK (56G8; Cell Signaling Technology), caspase-3 (Cell Signaling Technology), LC3 (Abgent, AJ1456c, San Diego, CA), SQSTM1 (p62) (5114, Cell Signaling Technology), Atg 5 (ab78073, Abcam, Cambridge, MA), Atg 9 (ab71795, Abcam, Cambridge, MA) and β-actin (4967, Cell Signaling Technology).

### Primary striatal cultures

Primary striatal cultures were carried out as described elsewhere [16]. Striata of fetal rats (embryonic day 17) from pregnant Sprague Dawley rats were dissected, and tissues were dissociated by repeated trituration with a pipette in PBS and 0.6% glucose. After decantation for 5 min, cells were collected by centrifugation at 1000*g* for 5 min. Cell pellets were resuspended in neurobasal medium supplemented with B27, glutamine, penicillin-streptomycin (Life Technologies, Grand Island, NY), and mercaptoethanol (Sigma-Aldrich, St. Louis, MO). Cells were seeded at 960 cells/mm^2^ into poly-D-lysine (Sigma)–coated 24-well plates. The cultures were maintained at 37°C in a humidified incubator with 5% CO_2_. On day 7, the medium was removed and replaced by fresh medium containing 3-NP at 1 mM. The cells were then cultured again at 37°C for the indicated time.

### Cell cycle assays

Cell cycle assay was performed as described previously [17]. For flow cytometric analysis, primary striatal cells were trypsinized, washed with PBS, and resuspended in ice-cold 80% ethanol. Briefly, 2.5 × 10^5^ fixed cells were incubated in 250 μL propidium iodide solution (500 mg/mL propidium iodide in 3.8 mol/L sodium citrate at pH 7.0) and 250 μL RNase A (10 mg/mL prepared in 10 mmol/L Tris–HCl at pH 7.5) for 30 min at 37°C in the dark. The stained cells were filtered through the cell strainer caps of Falcon polystyrene round-bottomed tubes. DNA content was analyzed on a FACScan (Becton Dickinson, San Jose, CA). Percentage of cells in each phase was determined using Cell Fit software (Becton Dickinson). Data was collected for at least 20,000 cells.

### Cell proliferation assay

WST-1 (Roche Diagnostics, Indianapolis, IN) was used to determine the effects of 3-NP on primary striatal cell proliferation according to the manufacturer’s protocol. Proliferation was calculated with respect to control cells and was tabulated using KaleidaGraph 3.0.1 (Synergy Software, Reading, PA) or Excel (Microsoft, Redmond, WA).

### HMGB1 knockdown by lentivirus

HMGB1-shRNA lentiviral plasmids were purchased from Applied Biological Materials Inc. (Richmond, BC, Canada). All recombinant lentiviruses were produced by transient transfection of 293T cells according to standard protocols. Briefly, subconfluent 293T cells were transduced with 20 μg of one of the two expression vectors, 15 μg of pAX2, and 5 μg of pMD2G-VSVG by calcium phosphate precipitation. After 16 h, the medium was changed, and recombinant lentiviral vectors were harvested twice, 24 and 48 h later. The raw viral supernatants were concentrated by polyethylene glycol precipitation. The primary striatal cells were transduced with comparable amounts of control-shRNA-expressing recombinant lentiviruses, or corresponding empty vector or control virus, in growth medium containing 6 μg/mL polybrene. Five days after transduction, the cells were subjected to puromycin selection.

### Statistical analysis

Differences between groups were analyzed by one-way analysis of variance (ANOVA) followed by Dunnett’s post hoc test. Differences were considered significant when p <0.05.

## Results

### 3-NP upregulated expression of HMGB1, p-JNK, and caspase-3 in vivo

**Fig 1:**
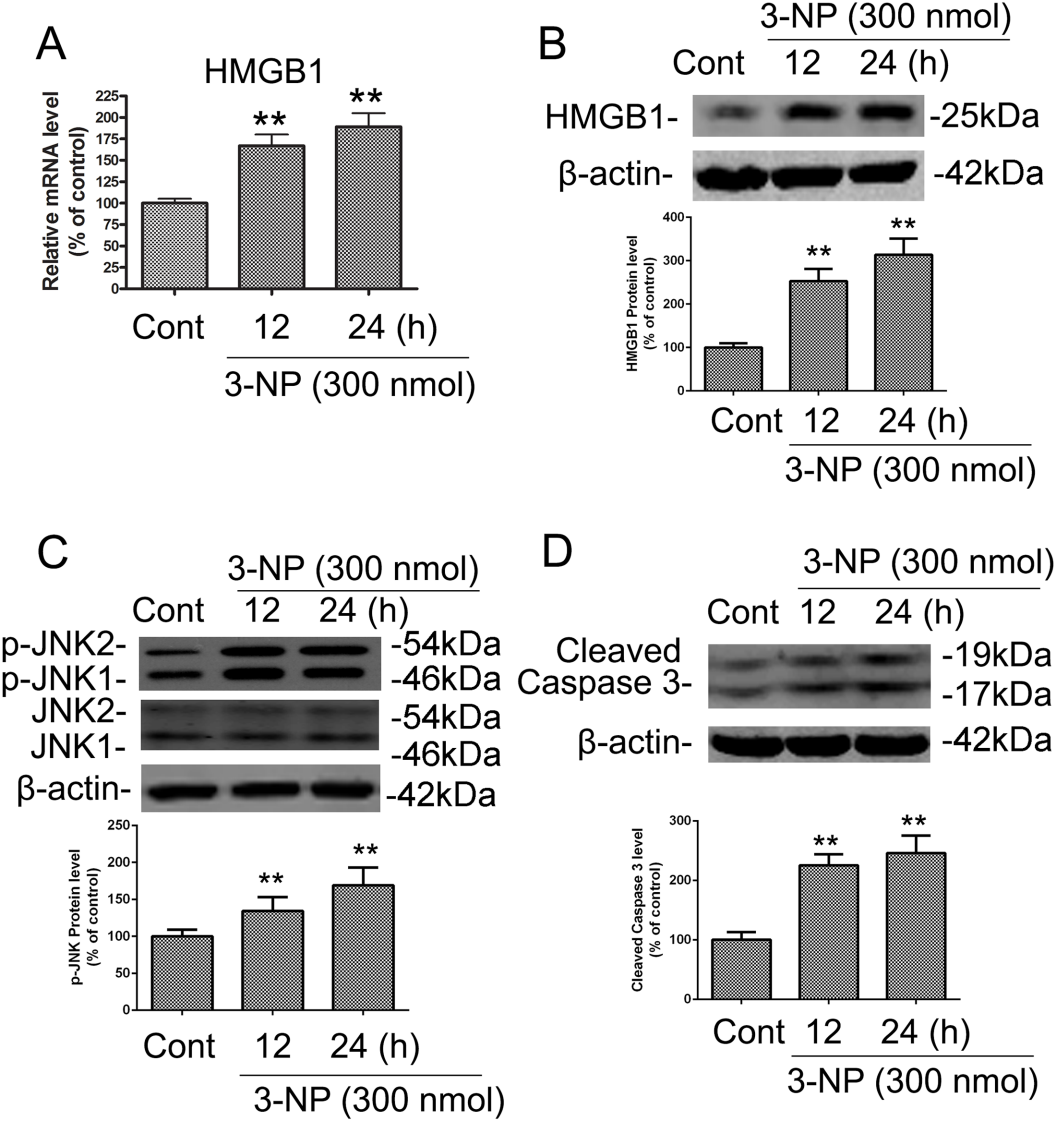
3-NP induced increases in the mRNA and protein levels of HMGB1 and in the protein levels of p-JNK and caspase-3 in rat striatum *in vivo*. The time-course of 3-NP–induced changes in HMGB1 expression, JNK phosphorylation, and caspase-3 cleavage was determined by isolating striatal tissue from rats that were killed 12 or 24 h after intrastriatal infusion of 3-NP (300 nmol) or vehicle control (CONT; isotonic saline solution, 1 μL). Striatal tissues were dissected for preparation of striatal extracts for immunoblotting. (A-B) 3-NP induced upregulation of the mRNA and protein levels of HMGB1. (C-D) 3-NP induced alterations in the levels of JNK phosphorylation and cleaved caspase-3. Densities of protein bands were analyzed with an image analyzer (Sigma Scan Pro 5) and normalized to the loading control (β-actin). Bars represent mean ± SE; n = 4 animals per group. Groups were compared by ANOVA followed by Dunnet’s post hoc test before data conversion. All panels, **p <0.01 vs control.

Our previous studies showed that 3-NP triggered p53-dependent activation of autophagy and cell death [7]. Due to an earlier report of interactions between HMGB1 and p53 as well as impact of p53 on HMGB1’s capacity to recognize DNA damage [10], we examined the effects of 3-NP on the expression of *HMGB1* mRNA and protein in the striatum. Intrastriatal injection of 3-NP in rats significantly increased levels of *HMGB1* mRNA and protein after 12 and 24 h (Fig 1A and 1B). It was shown that JNK modulation is an important molecular event in 3-NP–induced striatal degeneration [16] which led us to further investigate expression of p-JNK in striatal tissue after 3-NP exposure. Phosphorylation of JNK was increased after 12 and 24h post injection (Fig 1C) as well as enhanced activation of caspase-3, a key mediator of apoptosis signaling (Fig 1D). These results suggest that mitochondrial dysfunction induced by 3-NP triggered increased expression of HMGB1 and autophagy/apoptosis–relevant proteins.

### HMGB1 inhibitor glycyrrhizin decreased 3-NP–induced JNK phosphorylation, LC3-II and caspase-3 activation, and cell death *in vivo*

**Fig 2:**
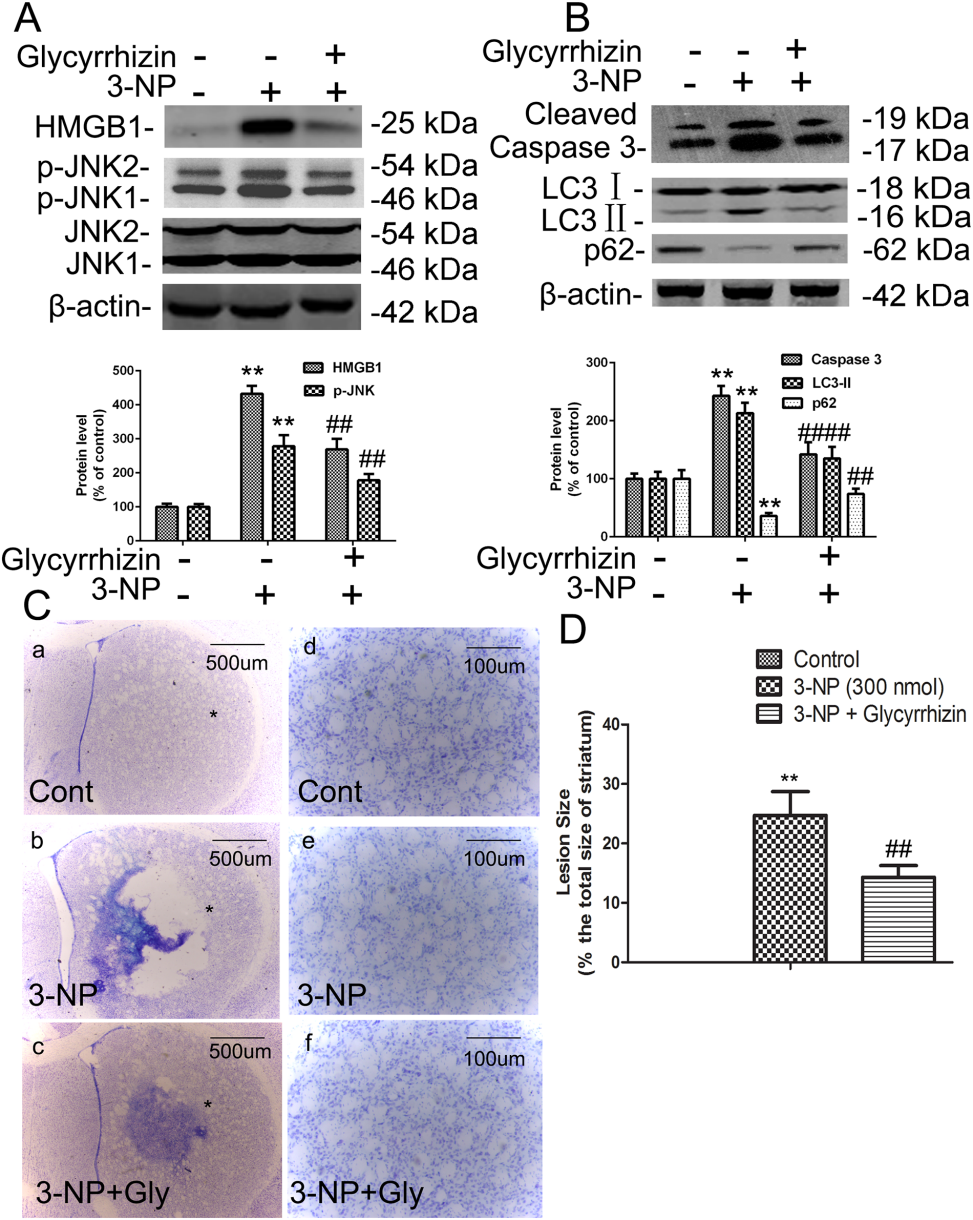
HMGB1 inhibitor glycyrrhizin inhibited 3-NP–induced upregulation of p-JNK and caspase-3 and death of striatal neurons *in vivo*. To study the effects of HMGB1 inhibition on phosphorylation of JNK, caspase-3 cleavage, and death of striatal neurons, striatal tissues were isolated from rats intrastriatally infused with glycyrrhizin (200 nmol) 1 h prior to 3-NP (300 nmol) infusion for an additional 24 h. (A-B) Glycyrrhizin inhibited 3-NP–induced increases in levels of p-JNK, LC3, SQSTM1, and cleaved caspase-3. (C) Cell death was identified by cresyl violet staining on brain sections from control and 3-NP–treated rats. Representative micrographs were taken in the center of the area injected with drugs (adjacent to needle tracks). (a and d) Vehicle treatment (CONT); (b and e) 3-NP treatment; (c and f) 3-NP + glycyrrhizin (Gly) treatment. Scale bars = 500 μm (a–c); 100 μm (d-f). (D) Quantitative analysis of the effects of glycyrrhizin on striatal lesions induced by 3-NP. Densities of protein bands were analyzed with an image analyzer (Sigma Scan Pro 5) and normalized to the loading control (β-actin). Bars represent mean ± SE; n = 3 samples per group. Groups were compared by ANOVA followed by the Dunnet’s post hoc test before data conversion. All panels, **p <0.01 vs control. ##p <0.01 vs. 3-NP alone.

To determine if 3-NP–induced HMGB1 expression is involved in activation of autophagy and apoptosis, we examined the effects of the HMGB1-specific inhibitor glycyrrhizin on JNK phosphorylation, expression of LC3-II and cleavage of caspase-3 in 3-NP–treated rats. LC3 is a mammalian homologue of yeast Apg8p, and LC3-II is required for the formation of autophagosomes and has been defined as a marker of autophagy in mammalian cells [18]. Glycyrrhizin has been shown to inhibit HMGB1-mediated signaling, including apoptosis [19]. Our results show that pretreatment with glycyrrhizin significantly blocked 3-NP–mediated elevation of p-JNK and decline of autophagy substrate SQSTM1 (p62), and reduced activation of autophagy marker LC3-II and apoptosis marker caspase-3 (Fig 2A and 2B). These results prompted us to examine the effects of glycyrrhizin on 3-NP–induced death of striatal cells by cresyl violet staining 14 days after 3-NP treatment. Lesion sizes were measured in four coronal sections from each striatum. While 3-NP alone caused substantial loss of striatal neurons and gliosis, glycyrrhizin pretreatment (100 nmol) significantly reduced the size of 3-NP–induced striatal lesions (p <0.05, n = 6; Fig 2C and 2D). These results suggest that HMGB1 is involved in 3-NP–mediated activation of the autophagy and apoptosis pathways that lead to striatal neuronal death.

### 3-NP upregulated expression of HMGB1, p-JNK, LC3 and SQSTM1 *in vitro* in primary striatal neurons

**Fig 3:**
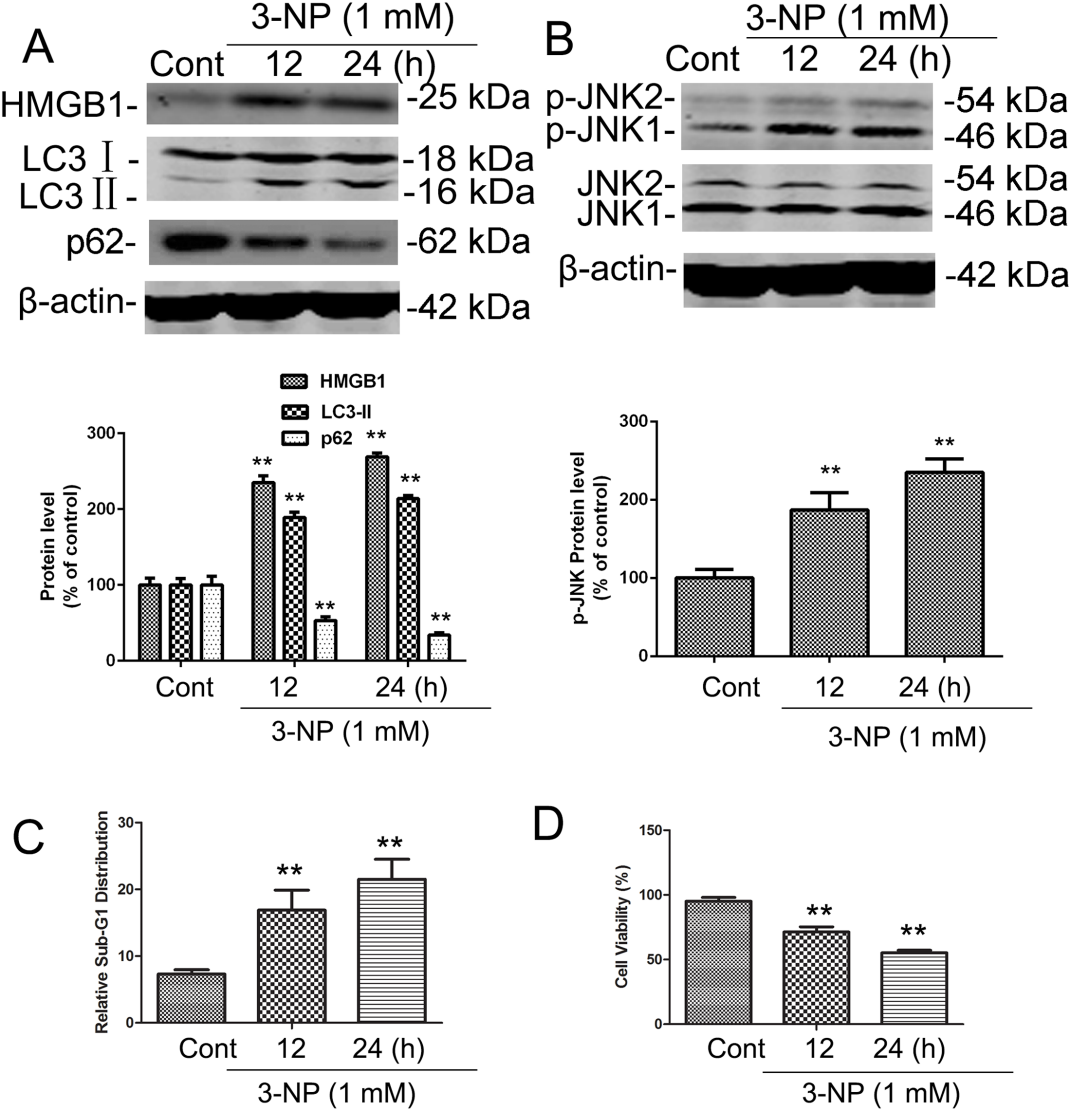
3-NP induced increases in the progressive activation of HMGB1, p-JNK, LC3, SQSTM1 and apoptosis in primary striatal neurons. Primary striatal neurons in culture were exposed to the indicated concentrations of vehicle (CONT) or 3-NP for 24 h. (A-B) Total cellular extracts were subjected to Western blotting for levels of HMGB1 and LC3-IIexpression, SQSTM1 degradation, and JNK phosphorylation (p-JNK). Equal loading was shown by β-actin. (C) Treated cells were subjected to cell cycle profiling, and the apoptotic sub-G1 fraction is shown. (D) Cell viability was evaluated by a tetrazolium dye–based assay, and percent viability was calculated relative to control values set to 100%. Results from three experiments are shown as mean ± SE; groups were compared by the Student *t*-test. All panels, ** p <0.01 vs. respective controls.

To elucidate the role of HMGB1 in 3-NP–induced striatal degeneration, we used a primary striatal neuron culture model. Time-course analysis of neurodegeneration after 3-NP (1 mM) treated cultured cells aimed to characterize autophagic and apoptotic features. In this model system, expression of HMGB1 and phosphorylation of JNK were significantly increased following 3-NP treatment for 12 and 24 h (Fig 3A and 3B). As a verification of autophagy activation, the present study demonstrated that 3-NP induced the elevation of LC3- II and the decline of SQSTM1 (Fig 3A). 3-NP increased neuronal apoptosis and cell death, as indicated by increases in the sub-G1 cell population and reduced cell proliferation (Fig 3C). Cell proliferation assay verified that NP-3 replicated neuronal death (Fig 3D). These results corroborate the effects observed *in vivo*, substantiating the involvement of HMGB1 in autophagy and apoptosis signaling.

### HMGB1 enhanced 3-NP–induced autophagy and apoptosis *in vitro*

**Fig 4:**
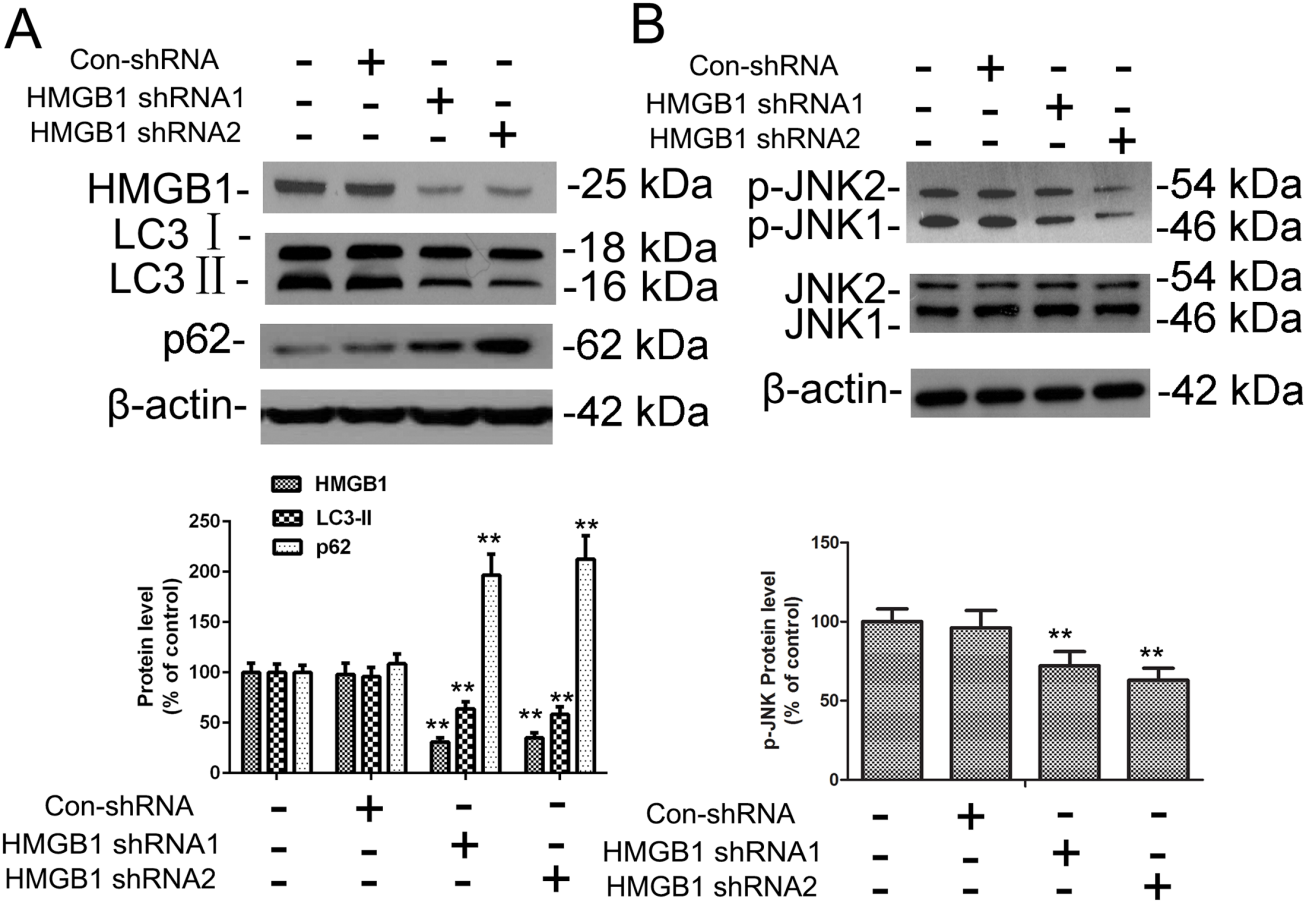
HMGB1 knockdown in primary striatal neurons without 3-NP treatment. (A,B) Primary striatal neurons were transduced with lentiviral vectors expressing either non-targeted control shRNA (Con-shRNA) or one of two different shRNAs targeting HMGB1 expression (HMGB1-shRNA1 and HMGB1-shRNA2) and cultured for 48 h. Total cellular extracts were subjected to Western blotting for HMGB1, LC3, SQSTM1, p-JNK, and JNK expression analysis. Densities of protein bands were analyzed with an image analyzer (Sigma Scan Pro 5) and normalized to the loading control (β-actin). Bars represent mean ± SE; n = 3 samples per group. Groups were compared by ANOVA followed by Dunnet’s post hoc test before data conversion. **p <0.01 vs control shRNA.

**Fig 5:**
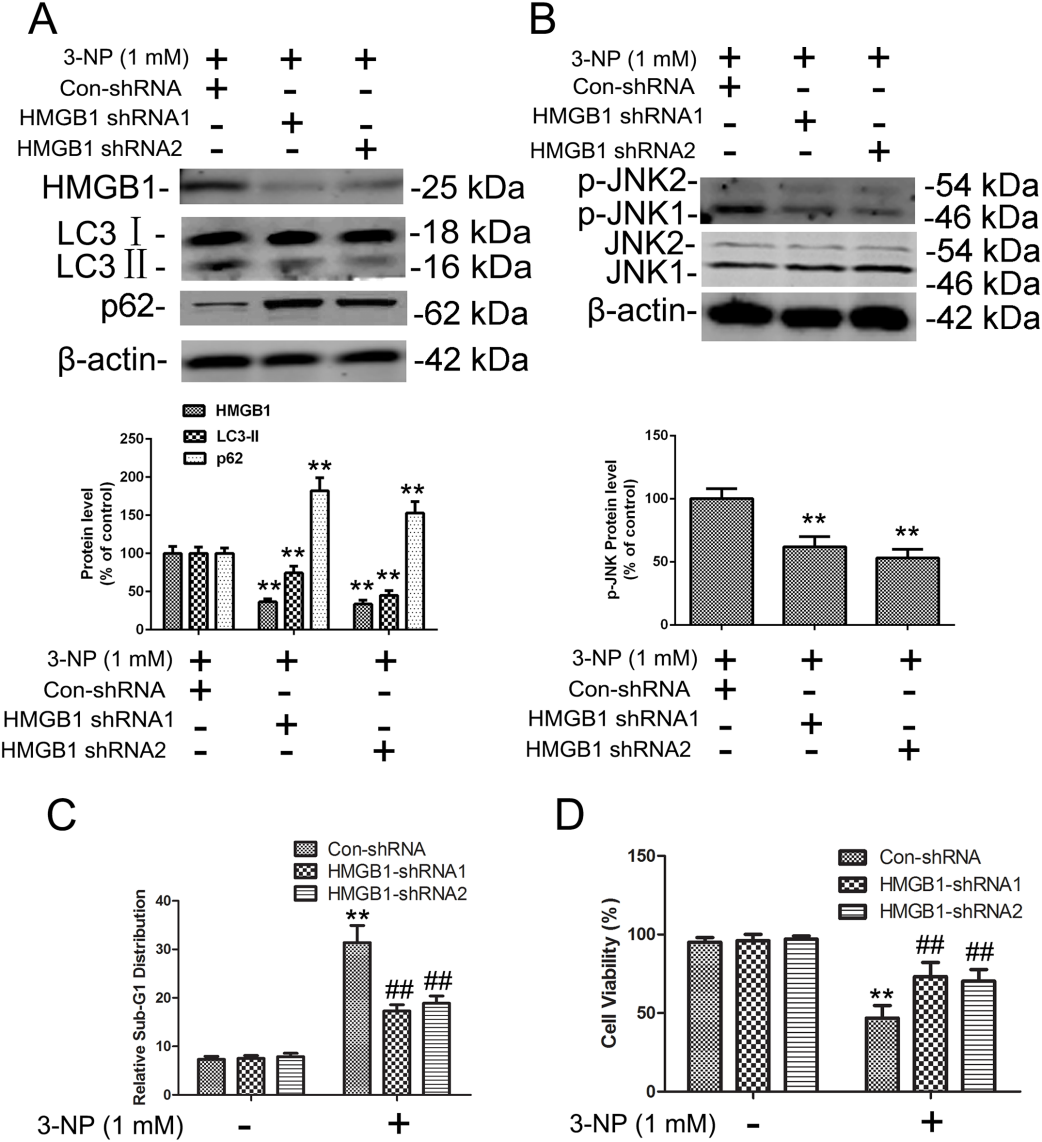
3-NP–induced expression of HMGB1, p-JNK, LC3, and SQSTM1 and apoptosis was reversed by HMGB1 knockdown in primary striatal neurons. Primary striatal neurons were transduced with lentiviral vectors expressing either non-targeted control shRNA (Con-shRNA) or one of two different shRNAs targeting HMGB1 expression (HMGB1-shRNA1 and HMGB1-shRNA2), then exposed to the indicated concentrations of vehicle or 3-NP for another 24 h. (A-B) Total cellular extracts were subjected to Western blotting for HMGB1, LC3, SQSTM1, p-JNK, and JNK expression analysis. Densities of protein bands were analyzed with an image analyzer (Sigma Scan Pro 5) and normalized to the loading control (β-actin). Bars represent mean ± SE; n = 3 samples per group. Groups were compared with ANOVA followed by Dunnet’s post hoc test before data conversion. **p <0.01 vs control shRNA. (C-D) Cells were treated with Con-shRNA or one of the two HMGB1 shRNAs, then subjected to cell cycle analysis and cell viability analysis as in Fig 3C and 3D. **p <0.01 vs control; ##p <0.01 vs. control shRNA.

To analyze the apparent impact of HMBG1 on autophagy and apoptosis signaling, we used a lentiviral shRNA approach to target HMGB1 expression in neuronal cells, including primary striatal neurons. Striatal neurons were infected by one of two HMGB1-targeting shRNAs or control shRNA followed by a selection for stable clones. Clones transduced with either of the HMGB1-targeted shRNAs had reduced expression of HMGB1 whether treated with 3-NP or not (Figs 4A and 5A). The decrease in LC3-II and increase in p62 after HMGB1 knockdown indicated that HMGB1 is involved in the regulation of basal autophagy without treatment of 3-NP. Similar to the incubation with the HMGB1 inhibitor glycyrrhizin, shRNA-mediated knockdown of HMGB1 expression significantly reduced 3-NP–mediated effects on autophagy and apoptosis as shown by reduced JNK phosphorylation, LC3-II activation, and decline of SQSTM1 (Fig 5A and 5B) with 3-NP treatment.

Because glycyrrhizin reduced the size of striatal lesions induced by 3-NP *in vivo*, we evaluated whether shRNA-mediated knockdown of HMGB1 would rescue primary striatal neurons from proapoptotic and antiproliferative effects of 3-NP. Neurons transduced with control shRNA or HMGB1 shRNA were treated with 3-NP (1 mM) and tested for apoptosis by determining the sub-G1 population as well as cell viability. Knockdown of HMGB1 protected primary neuronal cells from 3-NP–induced effects (Fig 5C and 5D). Together, these results indicate that HMGB1 has an important role in autophagy and apoptosis activation and promotes death of damaged primary striatal neurons.

**Fig 6:**
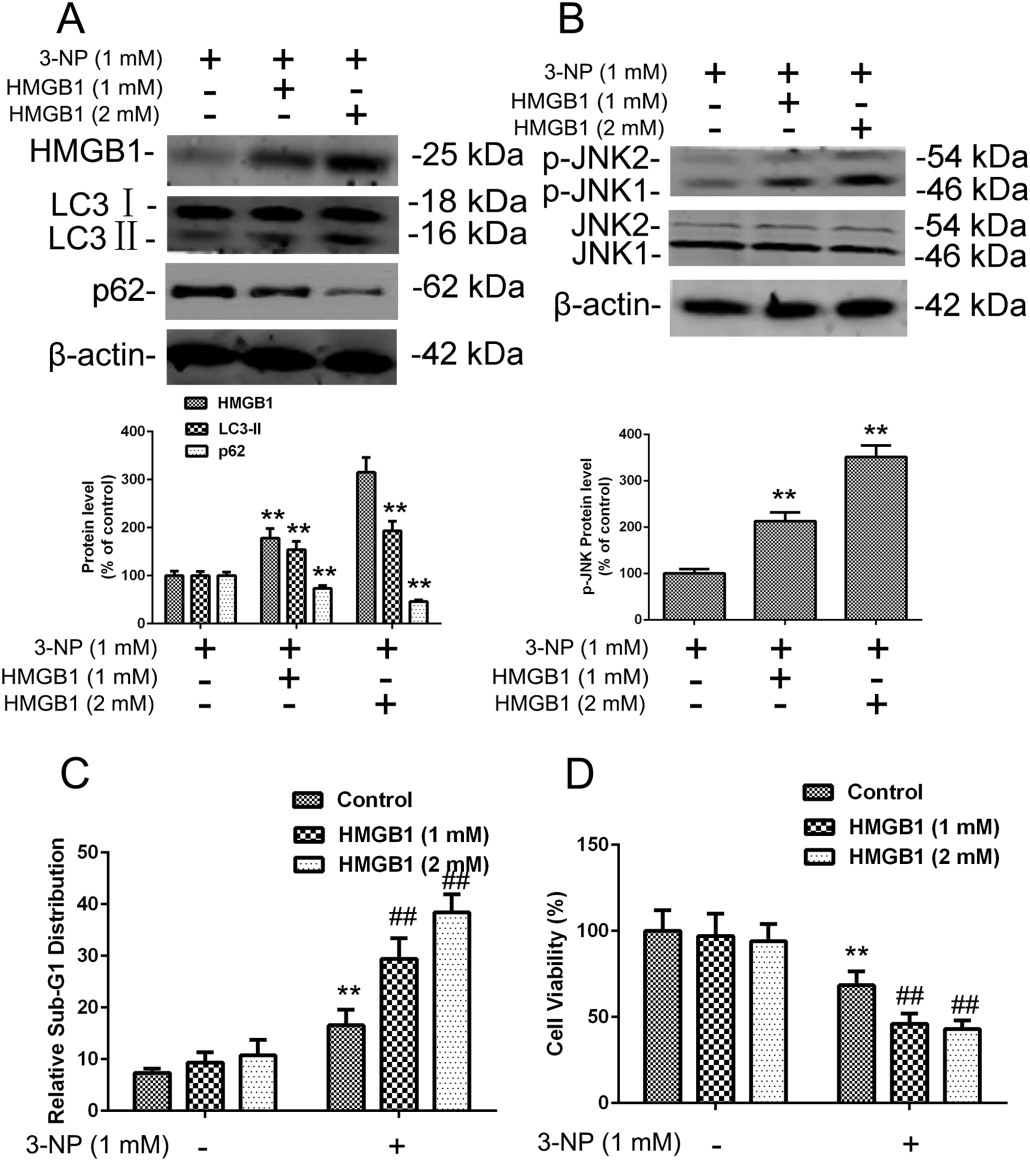
3-NP–induced expression of HMGB1, p-JNK, LC3, SQSTM1, and apoptosis were increased by exogenous HMGB1 treatment in primary striatal neurons. (A-B) Cells in culture were exposed to exogenous purified HMGB1 protein (1 or 2 mM) in the culture medium for 24 h followed by 24 h with added 3-NP. Total cellular extracts were subjected to Western blotting for HMGB1, LC3, SQSTM1, and JNK and p-JNK expression analysis. Densities of protein bands were analyzed with an image analyzer (Sigma Scan Pro 5) and normalized to the loading control (β-actin). Bars represent mean ± SE; n = 3 cell samples per group. Groups were compared by ANOVA followed by Dunnet’s post hoc test before data conversion. **p <0.01 vs control (C-D) Treated cells were subjected to cell cycle analysis and cell viability analysis as in Fig 3C and 3D. **p <0.01 vs control. ##p <0.01 vs. control.

To further characterize the role of HMGB1 in the regulation of 3-NP–induced autophagy and apoptosis, exogenous HMGB1 protein (1mM and 2mM) was added to the media for primary striatal neurons for 24 h prior to a 24 h lasting 3-NP treatment (S1 Fig)[12]. Similar to 3-NP treatment alone, this combined regimen elevated levels of HMGB1, resulting in elevation of LC3- II and JNK phosphorylation, and the decline of SQSTM1 (Fig 6A and 6B), further substantiating the role of HMGB1 in inducing autophagy signaling. Treatment with exogenous HMGB1 also increased cell death and decreased proliferation of primary neuron cells. Although increased levels of HMGB1 alone induced some apoptosis, treatment with 3-NP significant enhanced this apoptotic effect (Fig 6C and 6D). Next, we compared viability of control and HMGB1-treated primary striatal neurons, noting that the combination of HMGB1 with 3-NP decreased cell viability to a greater degree than 3-NPor HMGB1 alone (Fig 6D). These results suggest that exogenous expression of HMGB1 enhances autophagy and apoptosis activation and that these effects can be further enhanced by 3-NP treatment.

## Discussion

The results of this study suggest that mitochondrial dysfunction induced by 3-NP triggers a mixed form of cell death involving apoptosis, autophagy, and necrosis. Furthermore, suppressing HMGB1 blocked/reduced 3-NP–induced LC3-II activation, SQSTM1 degradation, and striatal cell death, whereas exogenous of HMGB1 treatment activated autophagy and apoptosis, however, where much stronger when combined with 3-NP The decrease in LC3-II and increase in SQSTM1 after HMGB1 knockdown further underscores a prominent role for HMGB1 basal autophagy signaling. These results reveal HMGB1 to be a key mediator in 3-NP–induced activation of autophagy and apoptosis in striatal neurons both *in vitro* and *in vivo*. These results presented here are also consistent with previous studies showing that 3-NP–induced cell death involves autophagy, apoptosis, and necrosis [7,20,21] and that HMGB1 and JNKs are critical regulators in autophagy activation [10,22].

Zhao *et al*. showed that exogenous HMGB1 functions as an autophagy effector by increasing transcriptional activation of the JNK and ERK pathways in human myeloid leukemia cells [13]. HMGB1 regulates JNK by binding to the autophagy regulator Beclin1 and subsequently regulating the formation of the beclin1–PI3KC3 complex that facilitates autophagic progression [23]. It was previously shown that Beclin1 binds to antiapoptotic Bcl-2 protein through its BH3 domain [24]. The JNK and ERK signaling pathways regulate Bcl-2 at three different sites in its BH3 and BH4 domains. Our results show that JNKs were activated by HMGB1 in response to mitochondrial dysfunction, which leads to the dissociation of Bcl-2 from Beclin-1. These studies suggested that HMGB1 regulates cell death via the JNK pathway.

Beclin1 is known to increase of LC3-II expression and autophagy pathway activation. Our previous study showed that autophagy inhibitors 3-MA and BFA attenuated autophagy activity and 3-NP–induced striatal cell death [7]. To support our conclusion that autophagy contributes to cell death in response to 3-NP treatment in striatal neurons, we examined the effect of 3-NP on the viability of primary striatal neurons in which Atg5 (autophagy protein 5) was knocked down. The finding that cell viability in 3-NP-treated cells was significantly reduced by the lack of Atg5 and Atg 9 (S2 Fig) supports the conclusion that autophagy regulating apoptosis plays an important role in cell death induced by mitochondrial insult. Impairments in mitochondrial energy metabolism are thought to be involved in most neurodegenerative diseases, and autophagy contributes to mitochondria dysfunction–induced neurodegeneration. Therefore, these finding suggests that the neuroprotective effect of HMGB1 inhibition is partially mediated through blocked autophagy signaling.

The specific sites for association of HMGB1 and autophagy, as well as the role of downstream located genes involved in mitochondrial dysfunction (i.e., 3-NP)–triggered cell death are still largely unknown. Livesey *et al*. indicated that HMGB1 and TP53 form a complex that regulates the balance between tumor cell death and survival[11], and a report from our group showed that TP53 target gene DRAM1 (damage-regulated autophagy modulator 1) regulated apoptosis by inhibiting BAX degradation[25]. Further studies on DRAM1 and BAX will be required to further elaborate on the detailed interaction between HMGB1 with autophagy and apoptosis signaling.

In summary, our data clearly indicate that activation of autophagy preceded the apoptotic process in striatal neurons and was mediated, at least partially, by HMGB1. Autophagy contributes to mitochondrial dysfunction–induced neurodegeneration. The blockage of both apoptosis and autophagy by inhibition of HMGB1 suggests that HMGB1 might be suitable target for a neuroprotective therapy for many neurodegenerative diseases.

## Supporting Information

S1 Fig HMGB1 exogenous treatment affected HMGB1 expression in primary striatal neurons. Cells were exposed to exogenous purified HMGB1 protein (1 or 2 mM) in medium for 48 h followed by analysis of HMGB1 proteins expression flow cytometry. Antibodies used were linked to allophycocyanin (APC)-HMGB1.
(TIF)

S2 Fig 3-NP–induced increases in activation of apoptosis were blocked by autophagy in primary striatal neurons. (A, B) Primary striatal neurons were transduced with lentiviral vectors expressing either non-targeted control shRNA (Con-shRNA) or one of two different shRNAs targeting Atg5 or Atg 9 expression (Atg5 or Atg 9-shRNA1 and Atg5 or Atg 9-shRNA2) for 48 h. Total cellular extracts were subjected to Western blotting for Atg5 expression analysis. Densities of protein bands were analyzed with an image analyzer (Sigma Scan Pro 5) and normalized to the loading control (β-actin). Bars represent mean ± SE; n = 3 samples per group. Groups were compared by ANOVA followed by Dunnet’s post hoc test before data conversion. **p <0.01 vs control shRNA. (C,D) Cell viability was evaluated by a tetrazolium dye–based assay, and percent viability was calculated relative to control values set to 100%. Results from three experiments are shown as mean ± SE; groups were compared by the Student *t*-test. **p <0.01 vs. respective controls.
(TIF)
