# Supplementary figures and images for: HMGB1 Promotes Mitochondrial Dysfunction–Triggered Striatal Neurodegeneration via Autophagy and Apoptosis Activation
Source: PLoS One. 2015 Nov 13;10(11):e0142901. doi: 10.1371/journal.pone.0142901 (PMC4643922; doi:10.1371/journal.pone.0142901)

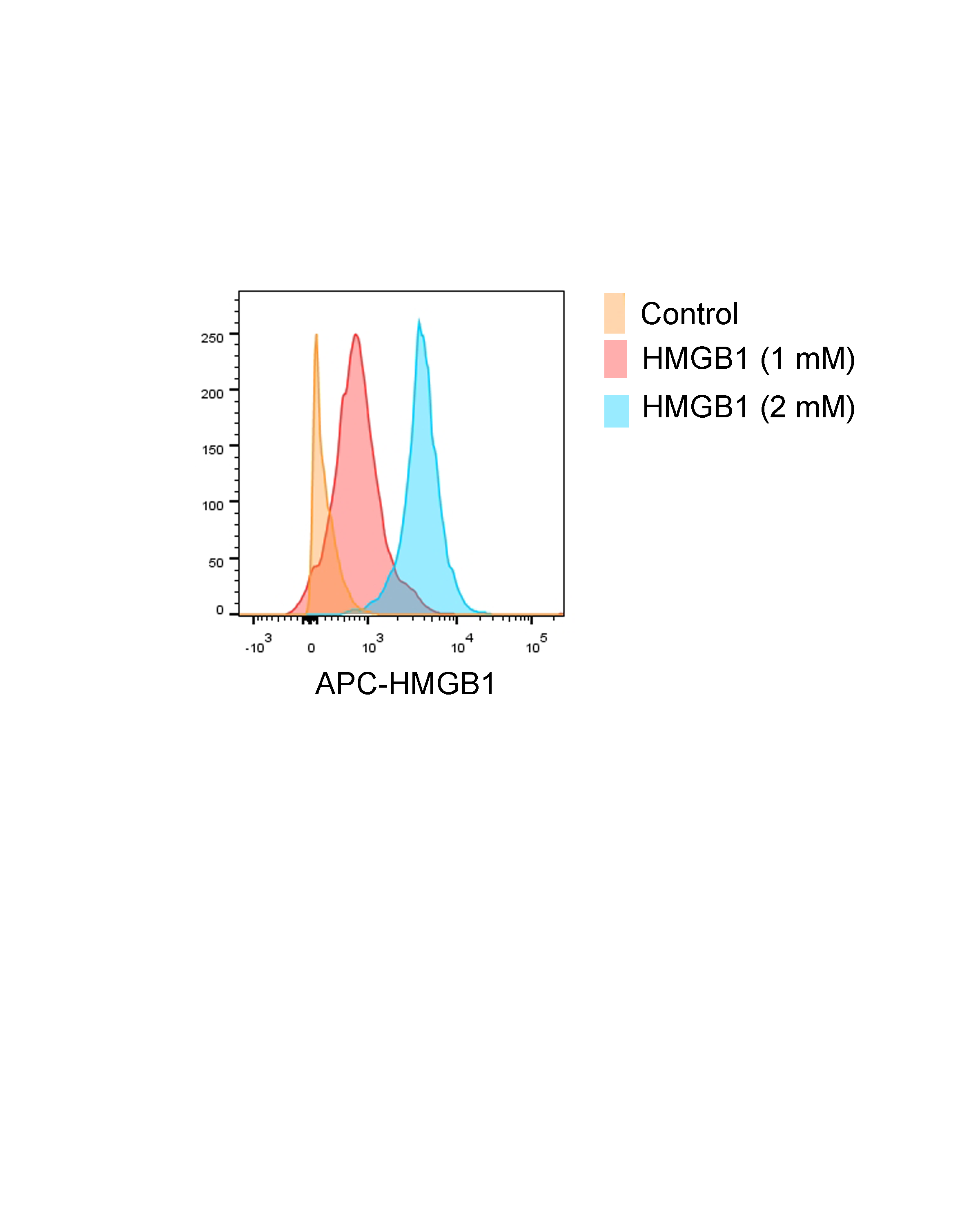

Supplement: S1 Fig — Cells were exposed to exogenous purified HMGB1 protein (1 or 2 mM) in medium for 48 h followed by analysis of HMGB1 proteins expression flow cytometry. Antibodies used were linked to allophycocyanin (APC)-HMGB1. (TIF) [file pone.0142901.s001.tif]

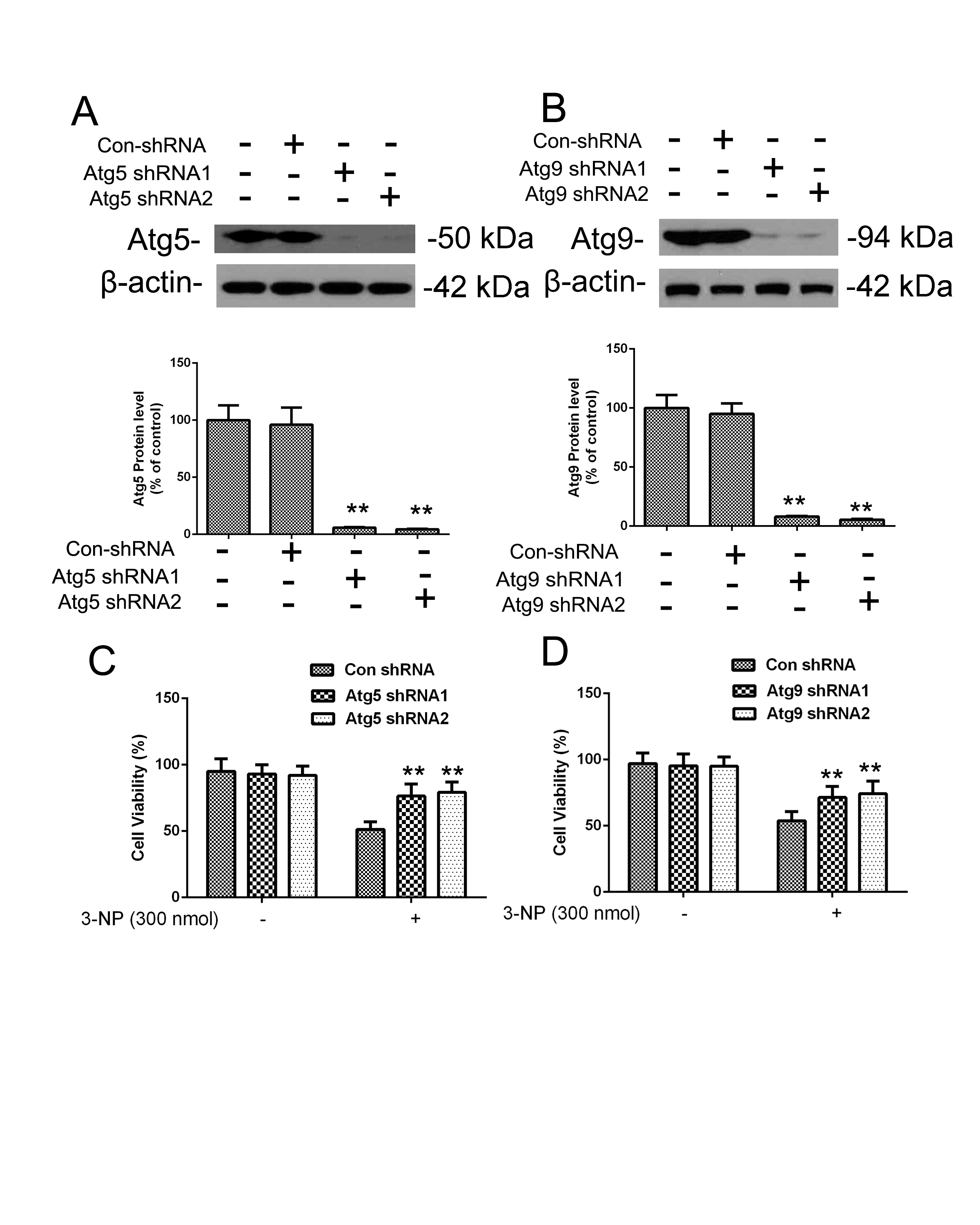

Supplement: S2 Fig — (A, B) Primary striatal neurons were transduced with lentiviral vectors expressing either non-targeted control shRNA (Con-shRNA) or one of two different shRNAs targeting Atg5 or Atg 9 expression (Atg5 or Atg 9-shRNA1 and Atg5 or Atg 9-shRNA2) for 48 h. Total cellular extracts were subjected to Western blotting for Atg5 expression analysis. Densities of protein bands were analyzed with an image analyzer (Sigma Scan Pro 5) and normalized to the loading control (β-actin). Bars represent mean ± SE; n = 3 samples per group. Groups were compared by ANOVA followed by Dunnet’s post hoc test before data conversion. **p <0.01 vs control shRNA. (C,D) Cell viability was evaluated by a tetrazolium dye–based assay, and percent viability was calculated relative to control values set to 100%. Results from three experiments are shown as mean ± SE; groups were compared by the Student t-test. **p <0.01 vs. respective controls. (TIF) [file pone.0142901.s002.tif]
